# “Not tonight zebrafish”: the effects of *Ruta graveolens* on reproduction

**DOI:** 10.1080/13880209.2017.1421234

**Published:** 2018-01-02

**Authors:** Mohammad Navid Forsatkar, Maryam HedayatiRad, Ana Carolina Luchiari

**Affiliations:** a Young Researchers and Elite Club, Karaj Branch, Islamic Azad University, Karaj, Iran;; b Young Researchers and Elite Club, Isfahan (Khorasgan) Branch, Islamic Azad University, Isfahan, Iran;; c Departamento de Fisiologia, Centro de Biociências, Universidade Federal do Rio Grande do Norte, Natal, RN, Brazil

**Keywords:** Rue, contraception, sexual behaviour, fertility, Danio rerio

## Abstract

**Context:** There is growing interest in the pharmacological evaluation of Rue due to its potential to treat a variety of clinical diseases. The plant seems to present potent endocrine disrupting effects, and its excretion and disposal are not a concern.

**Objective:** The effects of *Ruta graveolens* L. (Rutaceae) ethanol extract (RE) on reproductive behaviour, fertility, and steroid and thyroid hormone levels in zebrafish were investigated.

**Material and methods:** We exposed subjects to varying concentrations of RE, and one-tenth the LC_50_ concentration (2.37 ppm) was established as the sublethal dose. After 2 weeks exposure, reproductive behaviour, cumulative number of eggs laid, percentage of fertilized eggs, and whole body steroid and thyroid hormones were measured.

**Results:** Reproductive association behaviour did not differ between control and RE-exposed animals, but spawning attempts were reduced in RE exposed animals. Cumulative egg production between days 9 to 14, RE exposed fish laid 672 eggs while control fish laid 1242 eggs. Also, percentage of fertilized eggs was higher for the control than for the RE exposed fish. Estradiol-17β (E2) levels were reduced in females exposed to RE and testosterone (T) was statistically lower in both males and females treated with RE. Furthermore, thyroid hormones (T3 and T4) declined in fish treated with RE.

**Conclusion:** RE has endocrine disrupting potential in fish, which has important implications for studying the effects of unintentional pharmaceutical exposure. Moreover, the results demonstrate that drug exposure may affect more than just the overall level of behaviour, emphasizing the relevance of examining the effects of individual exposure. We reinforce the use of zebrafish as a model organism in physiology and behaviour, and raise concerns about the toxic effects of RE in non-target organisms such as aquatic vertebrates, which may ultimately affect human health.

## Introduction

Throughout human history, plants have been the basis of medical treatments. While many consider herbal infusions, ointment and balms alternative medicine, scientific evidence of active plant compounds contributes to increasing knowledge of traditional medicine. It is known that more than three thousand substances are used for human medical treatment (Fent et al. 2006). Several of these derive from plant extracts that, after being metabolized, are excreted with faeces or urine, entering the aquatic environment still bearing active molecules (Gaworecki and Klaine 2008). The concentration of these compounds in the environment has raised concerns about their potential for jeopardizing the biota (Daughton and Ternes 1999; Kolpin et al. 2002; Brooks et al. 2003).

One of the most widely used plants for medical purposes is rue, a perennial shrub native to the Mediterranean region, which is grown in many parts of the world (Asgarpanah and Khoshkam 2012). Of the two species of rue used for medical purposes, *Ruta graveolens* L. (Rutaceae), an odoriferous herb, is more important (Ratheesh and Helen 2007). Herbal medicines derived from *R*. *graveolens* extracts are increasingly used to treat a variety of clinical diseases; this plant has been shown to act as an antibiotic, cytotoxic (Ivanova et al. 2005), anti-inflammatory (Raghav et al. 2006), fungicide (Oliva et al. 2003; Meepagala et al. 2005), pain killer for rheumatism, and hypotensive (Chiu and Fung 1997). However, one of its most ancient prescriptions is related to its contraceptive and abortive effects (Gutiérrez-Pajares et al. 2003; Maurya et al. 2004; De Freitas et al. 2005). A number of phytochemical compounds have been identified in *R*. *graveolens*, including acridone alkaloids, coumarins, volatile substances, terpenoids, flavonoids, furoquinolines, saponins, tannins, glycosides and chalepensins (Kuzovkina et al. 2004; Hashemi et al. 2011). The last substance (chalepensin) has been suggested as the active component of the abortifacient (De Freitas et al. 2005). However, relatively little knowledge about rue’s mode of action is available, and while there is growing interest in the pharmacological evaluation of the plant, no attention has been given to excretion and disposal. Indeed, many pharmaceutical products are present in detectable levels in aquatic systems worldwide, some with endocrine disrupting effects on organisms (Kolpin et al. 2002; Blair et al. 2013).

As such, this study aimed at evaluating the toxic effects of *R. graveolens* extract, and testing its sublethal effects on reproductive behaviour, fertility, and steroid and thyroid hormone levels in zebrafish. In addition to the zebrafish’s advantages as an experimental model, such as small size, extra uterine development, transparent embryos, and short reproductive cycle, it is also a translationally relevant organism due to the relatively high degree of homology with the human genome (Feitsma and Cuppen 2008; Howe et al. 2013). Endocrine disruptors discarded in water bodies are a serious problem because they not only affect the life cycle and development of aquatic organisms, but may also impact human health as a consequence of bioconcentration. Thus, zebrafish sensitivity to discarded elements allows it to be used for both environmental monitoring and as physiological and behavioural screening for active compounds that may impact long-term health.

## Materials and methods

### Fish stock and maintenance

Adult zebrafish (*Danio rerio*, 5 months old, mixed sexes) were purchased from a local fish distributor and transferred to a storage system (140 L tanks). Animals were housed (one fish/L) with aerated and filtered water, and temperature (27 ± 1 °C), pH (7.3–7.8), hardness (180–245 mg/L as CaCO_3_), ammonia <0.1 mg/L, nitrite <0.05 mg/L, and dissolved oxygen (7.3–8.1 mg/L O_2_) were measured regularly. Fish remained under a 14:10 h light:dark cycle, with zeitgeber time (ZT) 0 corresponding to lights on time (8 am–10 pm). Zebrafish were fed twice a day (3% body weight) with BioMar dry pellets (42% protein, 22% fat, 1.8% fibres, 6.4% ashes) and frozen *Artemia*. Before the experimental procedures, male and female zebrafish were separated into two 140 L tanks for 7-days’ acclimation.

### Preparation of rue ethanol-extract (RE)

Aerial parts of *R. graveolens* were collected from the herbarium of the Faculty of Pharmacy, University of Tehran. Leaves were dried for 5 days at 40 °C. The extraction procedure was based on Forsatkar et al. (2016). Briefly, 50 g of dried crushed leaves were immersed in 500 mL of 70% ethanol under 48 h stirring. The solution was then filtered and lyophilized (Lyotrap, LTE Scientific Ltd), and the resulting powder (4.81 g) was stored in a dark, clean jar at room temperature (24 °C).

### LC_50_ value for RE

RE concentrations were prepared to determine the LC_50_ value of the plant using the static-renewal toxicity test, which is to expose the fish to a fresh solution of the same concentration every 24 h for 96 h, by replacing all the solution in the test tank every 24 h (Hoang et al. 2004; Belanger et al. 2013). Chronic exposure to the final concentration of 0, 5, 10, 15, 20, 25, 30 ppm was achieved by diluting the lyophilized *Ruta* powder (described above) in system water. We calculated these concentrations based on a previous study on *Artemia salina* L. and extrapolated to an approximate body mass of 650 mg of the weight of present zebrafish (Parra et al. 2001). This type of dosage selection is common when the appropriate taxon-specific dosage is unknown (e.g., Forsatkar et al. 2016). Solutions were prepared in 5 L glass beakers, which were used as fish tanks. For each RE concentration, 10 zebrafish (687 ± 71.35 mg) were added to each of the three beakers containing 5 L of the final RE solution. RE solutions were prepared fresh daily due to the unknown half-life of rue in water. For chronic RE exposure, beaker solution was changed every day to ensure appropriate water dosage of RE, a procedure that also allowed the desired RE concentration to be maintained in the beaker. The laboratory conditions were similar to what the fish experienced during the acclimation period (photoperiod: 14 h light: 10 h dark cycle, temperature (27 ± 1 °C), pH (7.2–7.8), hardness (165–270 mg/L as CaCO_3_), ammonia <0.1 mg/L, nitrite <0.02 mg/L, and dissolved oxygen (7–7.9 mg/L O_2_). Fish were exposed to RE for 96 h (4 days), 24 h per day and were not fed during the exposure procedure. Mortality was checked three times daily, and the dead fish removed.

Linear regression was used to calculate the 96 h LC_50_ values (GraphPad Prism 6) (Chen et al. 2011). The 96 h LC_50_ of RE in the adult zebrafish was 23.71 ppm. The sublethal RE concentration, one-tenth of the LC_50_ concentration (2.37 ppm) (Sprague 1971), was used to assess reproductive performance and steroid/thyroid hormone values in zebrafish.

### Fertility

A total of 36 females and 24 males were randomly selected from the stock, weighed (mean ± SD females = 7.13 ± 0.91 g and males = 5.58 ± 0.64 g) and assigned to one of two conditions: exposure to 0 or 2.37 ppm of RE for 2 weeks.

Males and females were kept isolated during the first 7 days of RE exposure (males in one tank and females in another tank). The control solution (system water) and the solution containing RE (lyophilized *Ruta* powder dissolved in system water) were prepared in 5 L glass tanks (30 × 10 × 20 cm; water height = 16.5 cm). Female zebrafish were held in groups of 6 in each tank, at the density of 1 fish/833 mL solution (control – 0 ppm RE: *n* = 3; treatment – 2.37 ppm RE: *n* = 3), and male zebrafish were kept in groups of 4 in each beaker, at the density of 1 fish/1250 mL solution (control *n* = 3, and treatment *n* = 3). Solutions containing the proper concentration of RE were prepared fresh daily and the volume of the beakers was completely replaced (every day at 9 am) to maintain appropriate water quality and desired RE concentration. Control beaker water was also replaced to equalize handling stress.

Groups containing 6 females and 4 males were formed on day 8. Thus, RE exposed males were netted and transferred to the RE exposed female beakers and the same procedure was performed for the control fish. During the procedure, the RE solution was replaced and a plastic mesh (4 × 4 mm) was added to each beaker, 3 cm from the bottom. Plastic mesh served as a porous barrier to prevent spawned eggs from being eaten by the adults. Fish from the two conditions were held in groups of 10/beaker for another 7 days; RE solution was replaced daily and spawned eggs were collected during water renewal (Lister et al. 2009). The eggs were transferred to a sterile glass Petri dish filled with clean, dechlorinated 27 °C water and the number of fertilized eggs was recorded 4 h later.

Fishes were fed twice a day with BioMar dry pellets and frozen *Artemia salina* during the 14-day testing period. No fish mortality was observed during the test.

### Whole-body hormone concentrations

The reproductive function is highly affected by the steroid hormones (Dittrich et al. 2011). Testosterone is the hormone related to spermatogenesis and sexual behaviour in males, while estradiol affects ovulogenesis and ovulation in females (Jannini et al. 1995; Krassas 2000). It is also known that the metabolic hormones produced by the thyroid impact sexual functioning by altering the steroid metabolism. Thus, the thyroid hormones disorders directly impact on fertility (Dittrich et al. 2011). In this sense, evaluating both steroid and thyroid hormones after RE exposure would give a thorough picture of the fertility disrupting effects of the extract.

Thus, zebrafish was exposed to RE for 14 days, and then 6 males and 6 females from each condition (2 males and 2 females from each beaker) were randomly chosen and used to measure testosterone (T) and estradiol-17β (E2) levels. Fish were not fed for 24 h before sampling. Steroid whole-body extraction was performed using the method described by Arukwe et al. (2008), with some modifications. Fish were euthanized with 500 mg/L of clove powder solution and immediately frozen at −20 °C. Each fish was then weighed, sliced into small sections, and a pool of two fish were minced and homogenized manually in 1:4 volume of phosphate buffered saline (PBS, pH 7.4) for 6 min on ice. Samples were then centrifuged at 2500 rpm for 4 min at 4 °C. The supernatant was transferred to a 10 mL screw top test tube and 5 mL of diethyl ether was added. The tube was vortexed for 1 min, immediately frozen at −20 °C for 2 h, and then the unfrozen portion was decanted into a fresh 10 mL test tube. The diethyl ether was evaporated under airflow, yielding a lipid layer containing the steroids. The extract was stored at −20 °C until ELISA was conducted on the samples. Enzyme immunoassays were run using commercial ELISA kits (PishtazTeb diagnostics, Tehran, Iran). To that end, the dry extract was reconstituted in 300 µL EIA buffer by vortexing, and concentrations of E2 and T were measured following manufacturer’s instructions. To measure each hormone, samples were analyzed in duplicate on a single plate. The inter-assay coefficients of variance for E2 and T were 3.06% and 4.41%, respectively. Whole-body steroid levels were determined using a 4-parameter sigmoid minus curve fit. Hormone concentrations were normalized to the weight (g) of the corresponding samples.

Whole-body thyroid hormone (THs) levels, triiodothyronine (T3) and thyroxine (T4) were measured by PishtazTeb diagnostics ELISA kits using the method described by Chen et al. (2012). To that end, another 6 fish from each condition (2 fish each from the control and RE extract treatment beakers) were randomly chosen, a male and a female pooled and homogenized in 0.5 mL ELISA buffer for 5 min by hand on ice. Samples were then vortexed vigorously for 10 min, and centrifuged at 5000 rpm for 10 min at 4 °C. The supernatants were collected and stored at −80 °C until analysis. The inter-assay coefficients of variance for T3 and T4 were 4.8% and 4.3%, respectively. Samples were analyzed in duplicate on a single plate.

### Sexual behaviour

Male zebrafish sexual behaviour was assessed after 14 days of RE exposure. Only males were evaluated because of the anti-androgenic properties of rue described in mammals and fish (Khouri and El-Akawi 2005; Forsatkar et al. 2016).

Twenty males (5.13 ± 0.74 g) from the stock population were divided into two groups: 10 fish were exposed to 2.37 ppm of RE and another 10 to 0 ppm of RE (control) in 5 L beakers for 14 days. Complete solution exchange was carried out daily for both groups. Fishes were fed twice a day with BioMar dry pellets and frozen *Artemia*.

On the 15th day, each male was individually placed into a transparent rectangular tank (30 × 10 × 15 cm; 10 cm water depth) and a non-exposed female (mature fish from the stock) was inserted into a separate partition of the tank. This procedure ensured that fish maintained visual and chemical contact but prevented physical interaction. The two fish were held in the tank overnight, and on the following morning, immediately after the lights were turned on, the barrier was lifted and the fish were allowed to have contact with each other. After 15 min of interaction, fish behaviour was recorded with a Sony handy camcorder (DSC-W800) for 15 min. Video recordings were converted into images (one image s^−1^) using a KM-media player and sexual behaviours in terms of female association and spawning attempts were counted. As described by Colman et al. (2009), female association refers to the male’s head alignment next to and slightly below the female’s head or anal fin, or a male swimming alongside a female for direct physical contact. Spawning attempt is defined as the number of quick oscillating tail movements the male exhibits against the female’s side to release sperm while the female releases eggs.

### Statistics

Data were analyzed using SPSS v22.0 software (SPSS, Chicago, IL). GraphPad Prism 6 was used to determine the LC_50_ value and prepare charts. We compared the number of eggs produced, number of fertilized eggs, levels of estradiol-17β (E2), testosterone (T), T3 and T4, as well as female association behaviour and spawning attempts between RE-exposed animals and control animals. For all these comparisons we used independent sample Student’s *t*-tests, since data were normally distributed according to the Kolmogorov–Smirnov test. A *p* value <0.05 was considered for statistical significance.

## Results

### LC_50_ value for RE

The value of LC_50_ for 96 h of RE exposure in adult zebrafish was 23.71 ppm. The limits 95% confidence (mg/L) was determined as 16.58-25.96, and the slope was 1.87.

### Fertility

**Figure 1.**
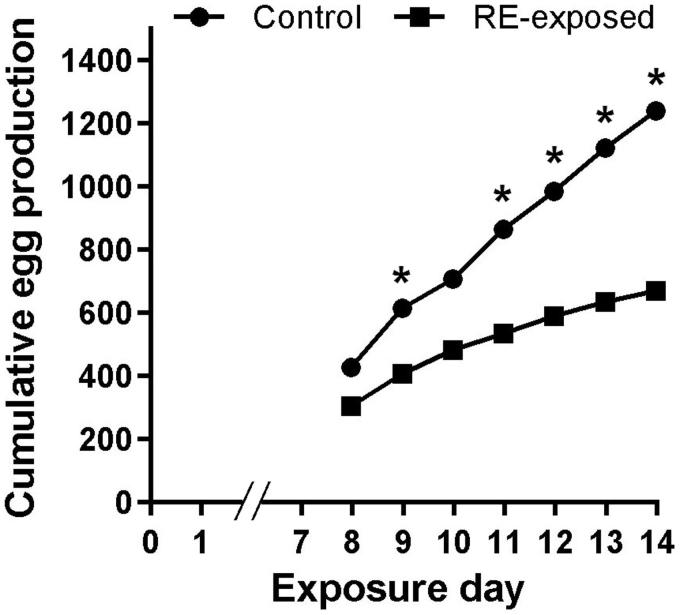
Zebrafish cumulative egg production following RE (rue extract) exposure. Male and female zebrafish were held for 7 days in 2.37 ppm of RE or control condition (blank water). Next, groups of 6 females and 4 males from both conditions (RE exposure and control) were formed and treatment continued for another 7 days, during which time spawned eggs were collected daily. The total number of eggs per day was compared between control and RE exposed groups using the Student’s *t* test. *indicates statistical difference at *p* < 0.05.

Zebrafish exposed to RE produced 672 eggs in one week, while control fish laid 1242 eggs within the same period (Figure 1). RE exposure significantly decreased the cumulative number of eggs by day 9 (t_4_ = 3.55; *p* = 0.024). There was also a significant difference in egg production between control and RE exposed fish on days 11 (t_4_ = 3.41; *p* = 0.027), 12 (t_4_ = 3.40; *p* = 0.027), 13 (t_4_ = 3.83; *p* = 0.018), and 14 (t_4_ = 9.001; *p* = 0.001) (Figure 1).

**Figure 2.**
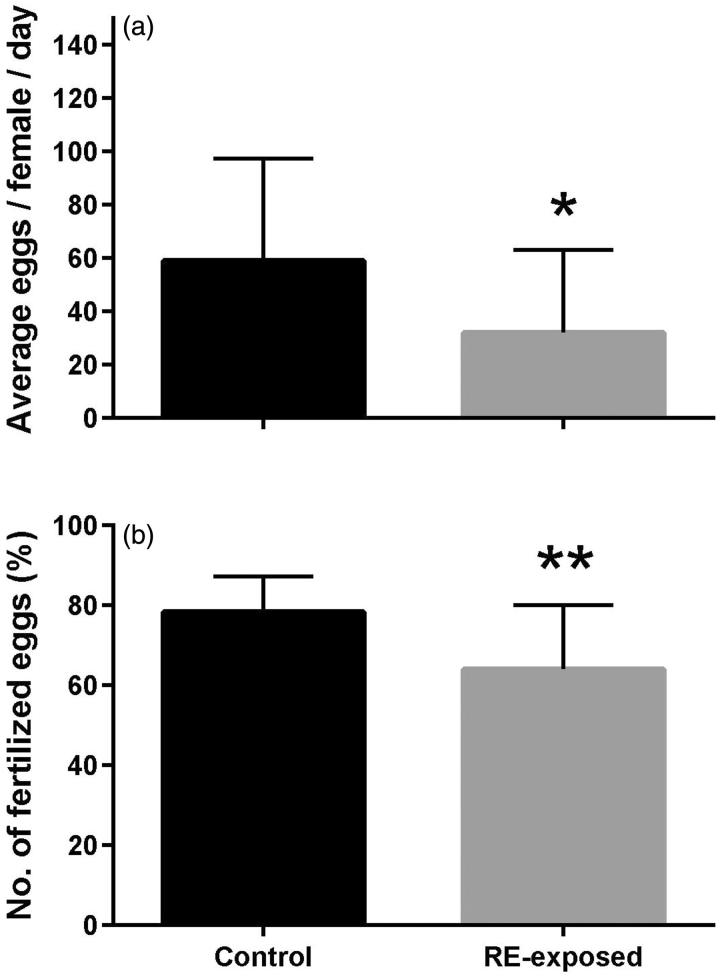
Zebrafish average number of eggs (a) and percentage of fertilized eggs (b) following rue extract (RE) exposure. Male and female zebrafish were held for 7 days in 2.37 ppm of RE or control condition (blank water) and then groups of 6 females and 4 males were formed. RE exposure continued for another 7 days, during which spawned eggs were collected daily. Data were compared between control and RE exposed groups using the Student’s *t*-test. *indicates statistical difference at *p* < 0.05 and **at *p* < 0.01.

Figure 2 depicts the average number of eggs laid and fertilized per female. There was a significant reduction in female egg production between control and RE-exposed zebrafish (t_40_ = 2.52, *p* = 0.016; Figure 2(a)). The percentage of fertilized eggs from the control was statistically higher than the RE exposed fish (t_40_ = 3.57, *p* = 0.001; Figure 2(b)).

### Whole-body hormone concentrations

Steroid (T and E2) and thyroid (T3 and T4) hormones were measured after 14 days of RE exposure.

**Figure 3.**
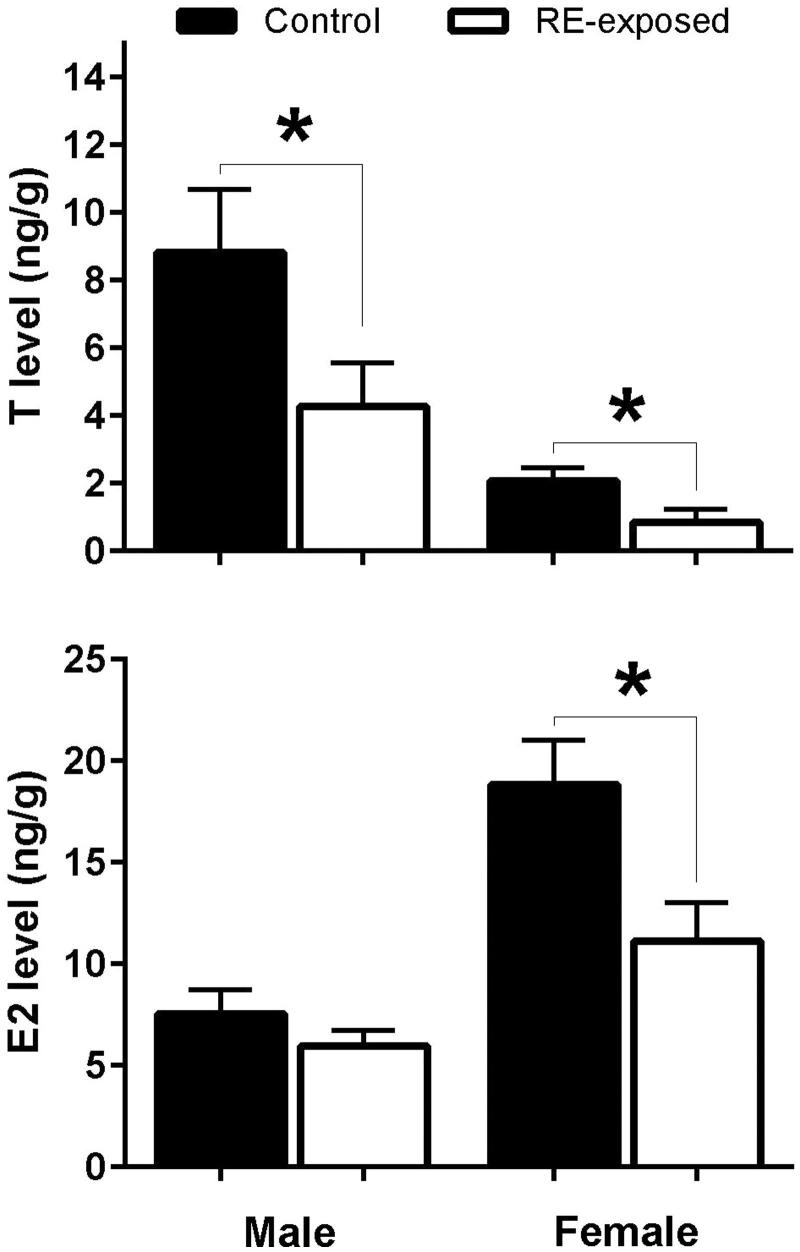
Whole-body concentration of steroid hormones: testosterone (a) and estradiol-17β (b) in zebrafish after rue extract (RE) exposure. Male and female zebrafish were held for 14 days in 2.37 ppm of RE or control condition (blank water). During the first 7 days, males and females were held separately and for the next 7 days, fish were held in groups of 6 females and 4 males. After 14 days of treatment, fish were sacrificed and total levels of testosterone and estradiol-17β were analyzed. Data from males and females were compared between treatments (RE and control) using the Student’s *t*-test. *indicates statistical difference at *p* < 0.05.

Total T level was statistically lower in both males and females treated with RE (males: t_4_ = 3.49, *p* = 0.025; females: t_4_ = 3.96, *p* = 0.017; Figure 3(a)). RE exposure also decreased the E2 level in females (t_4_ = 4.64, *p* = 0.01; Figure 3(b)), but not in males (t_4_ = 1.98, *p* = 0.118; Figure 3(b)).

**Figure 4.**
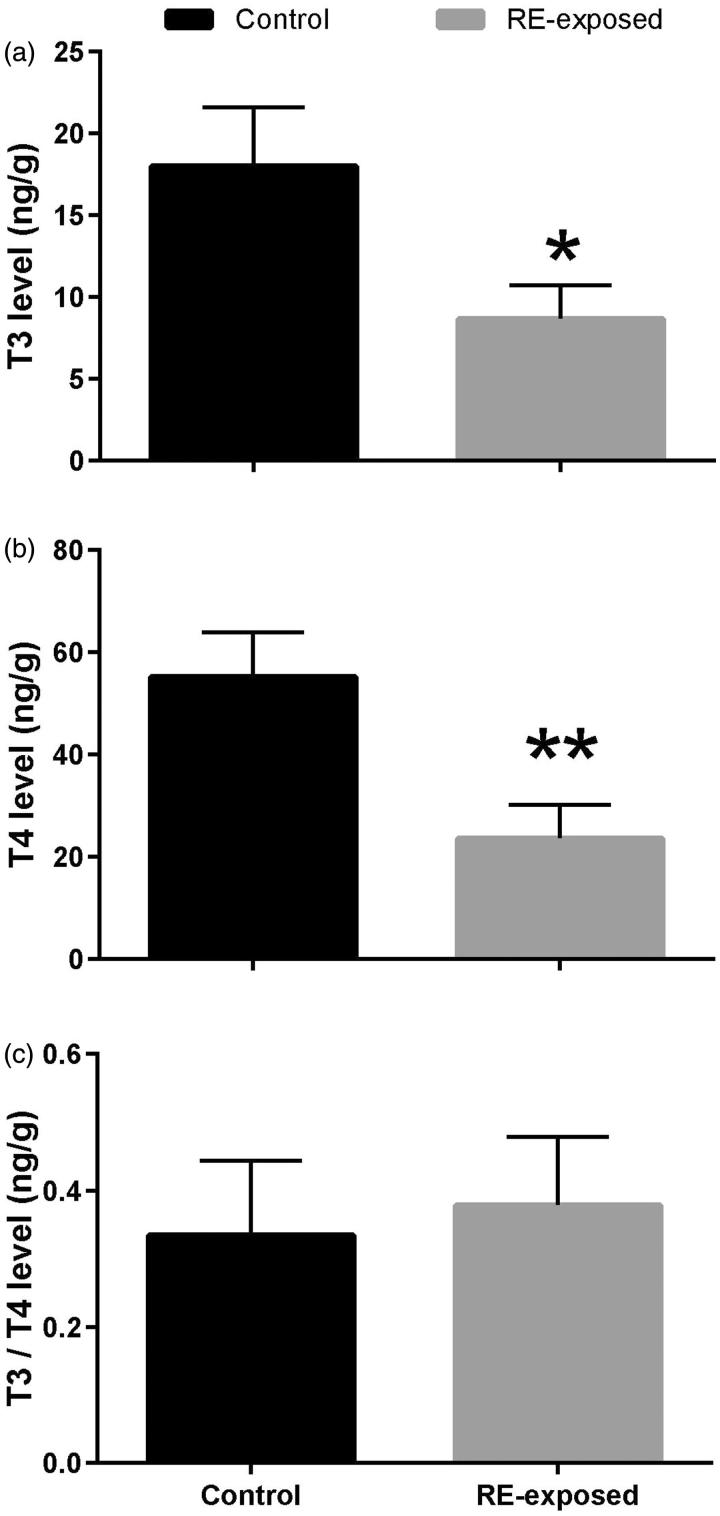
Whole-body concentration of thyroid hormones: triiodothyronine T3 (a), thyroxine T4 (b) and T3/T4 ratio (c) in zebrafish after rue extract (RE) exposure. Zebrafish were held for 14 days in 2.37 ppm of RE or control condition (blank water), after which total levels of thyroid hormones were analyzed. Data were compared between treatments (RE and control) using the Student’s *t-*test. *indicates statistical difference at *p* < 0.05 and **at *p* < 0.01.

Whole-body concentration of T3 was statistically lower in RE exposed fish (t_4_ = 3.88, *p* = 0.018; Figure 4(a)). The same results were observed for T4 level (t_4_ = 5.07, *p* = 0.007; Figure 4(b)). The T3/T4 ratio did not differ between control and RE-exposed animals (t_4_ = −0.51, *p* = 0.631; Figure 4(c)).

### Sexual behaviour

**Figure 5.**
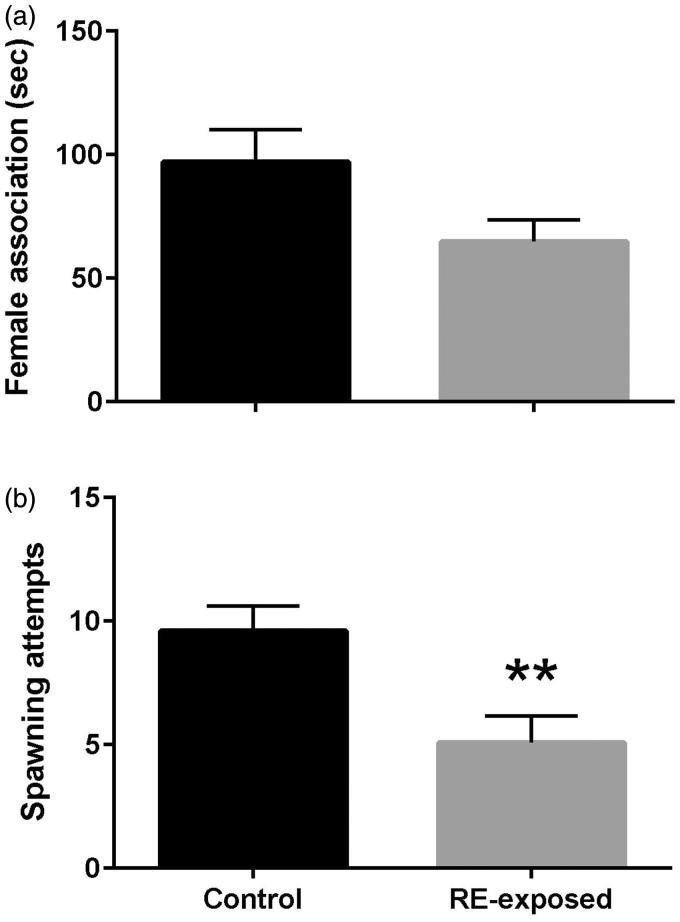
Sexual behavior in terms of female association (a) and spawning attempts (b) in zebrafish exposed to rue extract (RE). Male and female zebrafish were held for 14 days in 2.37 ppm of RE or control condition (blank water), after which one male and one female were paired and behaviour was observed for 15 min during the first hour of daylight. Female association occurs when the male aligns his head with the female’s head or anal fin, with direct physical contact. Spawning attempt refers to the number of tail oscillations the male shows during sperm release while the female releases her eggs. Data were compared between treatments (RE and control) using the Student’s *t*-test. **indicates statistical difference at *p* < 0.01.

Following 14 days of RE exposure, couples of zebrafish were evaluated in terms of female association and spawning attempts. Association behaviour did not differ between control and RE-exposed animals (t_18_ = 2.046, *p* = 0.056; Figure 5(a)). However, spawning attempts declined in RE exposed animals (t_18_ = 3.087, *p* = 0.006; Figure 5(b)).

## Discussion

Endocrine disruptors that enter the environment have the potential to affect the life cycle and development of non-target organisms, disrupting normal functioning and interfering in physiological and behavioural responses. This study examined how exposure to *Ruta graveolens* extract (RE) influences reproductive behaviour, fertility, and steroid and thyroid hormone levels in zebrafish. We observed that RE exposure decreases both steroid (testosterone and estradiol) and thyroid (triiodothyronine and thyroxine) hormone levels, reduces spawning attempts and lowers the number of eggs spawned and fertilized.

The effects of RE on egg production and fertilization seem to be related to its action on gonadal steroids. It is well established that control of reproductive activities is mediated by the endocrine system, whereby each hormone plays a specific role in different stages of the reproductive process (Kobayashi et al. 2002). Sex steroids are required for spermatogenesis and spermiation, sexual differentiation, and sexual maturation (Devlin and Nagahama 2002; Schulz et al. 2010), and are strongly involved in the behavioural repertoire associated with reproduction (Dey et al. 2010). Thus, gonadal steroid levels may affect the quality/quantity of gonadal products, both eggs and sperm, during gametogenesis (Kime 1998). In this respect, RE exposure seems to disrupt the biological pathways involved in the production of sex hormones, either through steroidogenic enzyme modifications (for example: aromatase) or via indirect effects on feedback loops (Mills and Chichester 2005), leading to inhibition of egg production and/or sperm release.

Other studies investigating rue have shown adverse effects on the aggressive and reproductive behaviour of mammals. For instance, rue extract decreased territorial defence and sexual behaviour in male Albino rats (Khouri and El-Akawi 2005), and hampered preimplantation embryo development in mice (Gutiérrez-Pajares et al. 2003). Moreover, rue extract exerted immobilization effects on human sperm (Harat et al. 2008). In fish, however, there is only one report of rue’s diminishing effect on agonistic and reproductive behaviour, and on the number of spermatozoa in male *Betta splendens* (Forsatkar et al. 2016). Thus, our study of RE effects in zebrafish, considered a translational model, contributes to current knowledge and reinforces ideas that may be useful to physiologists and clinicians in terms of the potential of rue as a pharmaceutical that disrupts conception.

In our study, we report that not only steroid hormones but also the production of thyroid hormones were inhibited in zebrafish in response to RE exposure. The metabolic, reproductive and behavioural functions of thyroid hormones are well documented in fish (reviewed in Carr and Patiño 2011). Gonadal development and reproductive activity of various teleost species depend on the proper functioning of the thyroid, and as such, any factors that alter thyroid functioning may disrupt reproductive processes or outcomes (e.g., Mukhi and Patiño 2007; Carr and Patiño 2011). Thus, the decline in egg production and fertilization may be attributed to the effects of RE exposure on gonadal hormones, thyroid hormones or both at different levels. The mechanism of action and potential means by which RE causes its effects has yet to be investigated, and even though we are paving the way in this direction, more research is needed. It is known that by interfering with thyrotropin stimulating hormone receptors (TSHr; Boas et al. 2012), or by inhibiting enzyme activity (such as iodothyronine 5’-deiodinase (Chaurasia et al. 1996) and thyroid peroxidase (Thomas 2008)), chemicals may disturb overall thyroid gland activity. Moreover, rue extract was shown to have hepatotoxic properties (El Agraa et al. 2002) related to thyroid dysfunction (e.g., Messarah et al. 2010). However, neurophysiological regulation of thyroid hormones or hepatic activity in fish were not investigated in the present study, allowing us only to hypothesize the possible ways in which RE exposure may have affected T3 and T4 levels. Indeed, numerous studies have demonstrated that drugs entering the environment affect TH levels in different species (reviewed in Carr and Patiño 2011).

In this study, we observed the effects of RE exposure on both hormone levels and behavioural expression. Sexual behaviour is the ultimate arbiter of animal reproductive output (Huang et al. 2015). Behaviour, in turn, depends on the intricate relationship between molecular, cellular and physiological responses that can all (or only partially) be affected by environmental pollutants (Saaristo et al. 2010; Huang et al. 2015). Thus, by measuring the behaviour involved in reproduction, one can evaluate aspects of physiological functioning. To date, little is known about the effects of RE on fish behaviour; however, we present data suggesting that RE disrupts gonadal and thyroidal functioning, which ultimately reflects on the sexual and reproductive performance of zebrafish. Our results corroborate those of other studies that have reported decreased sexual behaviour related to reduced levels of T (Toft and Guillette 2005) and THs (Mukhi and Patiño 2007) in fish exposed to water contaminants, and suppression of sexual behaviour in rats treated with rue extract (Khouri and EL-Akawi 2005).

Overall, our results indicate RE’s potential as an endocrine disrupting chemical in fish, and raise concern about its toxic effects in non-target organisms such as aquatic invertebrates and vertebrates, which may ultimately affect human health. While there is still no report about the environmental concentrations of rue, precluding extrapolation of these results to environmental levels, the lack of information is partly related to the fact that rue is an herb traditionally used in developing countries (Harat et al. 2008; Freire et al. 2010) where the hazardous impacts of chemicals on nature are still not a concern. To overcome this limitation, we used the standard approach to determine LC_50_, and one-tenth the LC_50_ value as the sublethal concentration to be tested. The mortality of zebrafish due to RE exposure in the present study suggests the toxic action of RE in physiological processes such as enzyme activity (Khouri and EL-Akawi 2005), liver functioning and haematological patterns (Freire et al. 2010). In summary, we have shown that RE negatively affects the levels of key hormones involved in reproduction (gonadal and thyroid hormones) and ultimately disrupts egg production and fertilization in zebrafish. Thus, the endocrine disrupting properties of *R*. *graveolens* could link physiological functions and behavioural aftereffects in a translational model. However, further studies on this issue are needed to better understand the effects of RE on other relevant aspects regarding the proper functioning of physiology and behaviour.
